# Private Medical Teaching

**Published:** 1846-07

**Authors:** 


### PRIVATE MEDICAL TEACHING.

Dr. Gordon, of Clinton College, Tennessee, proposes to give instruction to students of medicine next winter, preparatory to their attendance upon lectures in the public schools. Anatomy and chemistry will be taught in the dissecting room and laboratory, and lectures will be delivered on physiology. The session will commence and close with the lectures in the medical schools. All who know Dr. Gordon's industry and fondness for teaching will look for good students from him. His enterprise commends itself to his young neighbors who are about commencing the study of medicine.

